# The First Reported Case of Hybrid Argon Plasma Coagulation Ablation for Symptomatic Cervical Inlet Patches Refractory to Proton Pump Inhibitor Therapy

**DOI:** 10.5152/tjg.2026.25581

**Published:** 2026-03-08

**Authors:** Yoen Young Chuah, Yeong Yeh Lee, Ping-Huei Tseng, Shih-Peng Hsieh, Chu-Kuang Chou, Yow-Ling Shiue

**Affiliations:** 1 Department of Biological Sciences, National Sun Yat-sen University, Kaohsiung, Taiwan; 2 Division of Gastroenterology and Hepatology, Department of Internal Medicine, Ping Tung Christian Hospital, Pingtung, Taiwan; 3 Department of Medicine, Universiti Sains Malaysia School of Medical Sciences, Kota Bahru, Malaysia; 4 Division of Gastroenterology and Hepatology, Department of Internal Medicine, National Taiwan University Hospital, Taipei, Taiwan; 5 Department of Pathology, Ping Tung Christian Hospital, Pingtung, Taiwan; 6 Division of Gastroenterology and Hepatology, Department of Internal Medicine, Ditmanson Medical Foundation Chia-Yi Christian Hospital, Chiayi, Taiwan; 7 Institute of Biomedical Sciences, National Sun Yat-sen University, Kaohsiung, Taiwan

Dear Editor,

Cervical inlet patch (CIP) comprises islets of heterotopic gastric columnar mucosa in the proximal esophagus, with a reported incidence ranging from 1% to 12% on routine esophagogastroduodenoscopy.^1^^,^^2^ Although frequently incidental, CIP has been associated with clinically relevant laryngopharyngeal symptoms, including globus sensation, hoarseness, throat clearing, burning throat, and dysphagia, in a subset of patients.^3^^,^^4^ Recent data further support an association between CIP and dysphagia, highlighting its potential clinical relevance.^5^ Acid suppression with proton pump inhibitors (PPIs) is commonly prescribed; however, symptom persistence despite adequate PPI therapy is not uncommon.

Endoscopic ablation using argon plasma coagulation (APC) has been the most established interventional treatment for symptomatic, PPI-refractory CIP, demonstrating symptom improvement in over 80% of patients and durable long-term efficacy with a low complication rate in Western countries.^6^^,^^7^ In contrast, data from Asian populations are limited, with only a single published case report to date reporting comparable symptomatic improvement following APC ablation.^8^ Radiofrequency ablation (RFA), although evaluated only in small prospective cohorts, has also shown promising short- to mid-term symptomatic and histologic outcomes.^9^ Hybrid argon plasma coagulation (hybrid APC), which combines submucosal fluid injection through a waterjet system to create a protective cushion before ablation, has been proposed as an alternative treatment for CIPs with less risk of causing post-procedure stricture.^10^ However, to date, hybrid APC has not been specifically described for the treatment of symptomatic CIP in the literature.

We report the case of a 46-year-old man with persistent laryngopharyngeal reflux–like symptoms refractory to medical therapy. Informed consent was obtained from the patient. He had received pantoprazole 40 mg once daily for four weeks, without symptomatic improvement. His baseline Visual Analog Scale (VAS, 0-10) scores prior to PPI therapy were as follows: dry throat 6, burning throat 6, globus sensation 8, hoarseness 7, and throat clearing 8. After PPI therapy, symptoms persisted or worsened (dry throat 8, burning throat 8, globus sensation 7, hoarseness 8, throat clearing 8).

Esophagogastroduodenoscopy revealed two well-demarcated, salmon-colored patches measuring 0.8 cm at 18 cm and 0.4 cm at 15 cm from the incisors (Figure 1A and B). Histopathologic examination demonstrated ectopic gastric-type mucosa adjacent to native esophageal squamous epithelium, with prominent foveolar hyperplasia and elongated glands, consistent with gastric heterotopia (cervical inlet patch) (Supplementary Figure 1).

Given the persistance of symptoms and the patient’s preference, cap-assisted hybrid APC was performed using pulsed APC settings of 60 W and 2 L/min, following submucosal saline injection to create a protective cushion. The ablation was conducted using an ablate–clean–ablate technique, with 30 applications for the 0.8 cm lesion and 20 applications for the 0.4 cm lesion, achieving a near-complete macroscopic ablation in the initial session (Figure 1C and D). No immediate or delayed adverse events, including bleeding, ulceration, or stricture, were observed.

At the 1-week follow-up, the patient reported marked improvement in symptoms (dry throat 1, burning throat 0, globus sensation 4, hoarseness 1, throat clearing 2). Six-month follow-up endoscopy demonstrated a tiny residual patch (~0.2 cm) at 18 cm, which was successfully treated with conventional APC (Supplementary Figure 2A and B). Symptom control remained durable at 6 months (dry throat 1, burning throat 0, globus sensation 2, hoarseness 1, throat clearing 2), with no evidence of stricture formation.

This case illustrated the potential role of hybrid APC as a safe and effective treatment option for PPI-refractory symptomatic CIP. The novelty of this report lies in the use of a hybrid APC device, which enables submucosal fluid injection prior to ablation—an approach not previously reported for this indication. Compared with conventional APC, hybrid APC may offer improved safety through controlled ablation depth, which is particularly relevant in the cervical esophagus. While RFA remains a promising alternative, APC continues to be the most widely studied modality, and hybrid APC represents an emerging technique that warrants further prospective evaluation. Larger studies with longer follow-up are required to define its comparative efficacy and long-term outcomes.

## Supplementary Materials

Supplementary Material

## Figures and Tables

**Figure 1.**
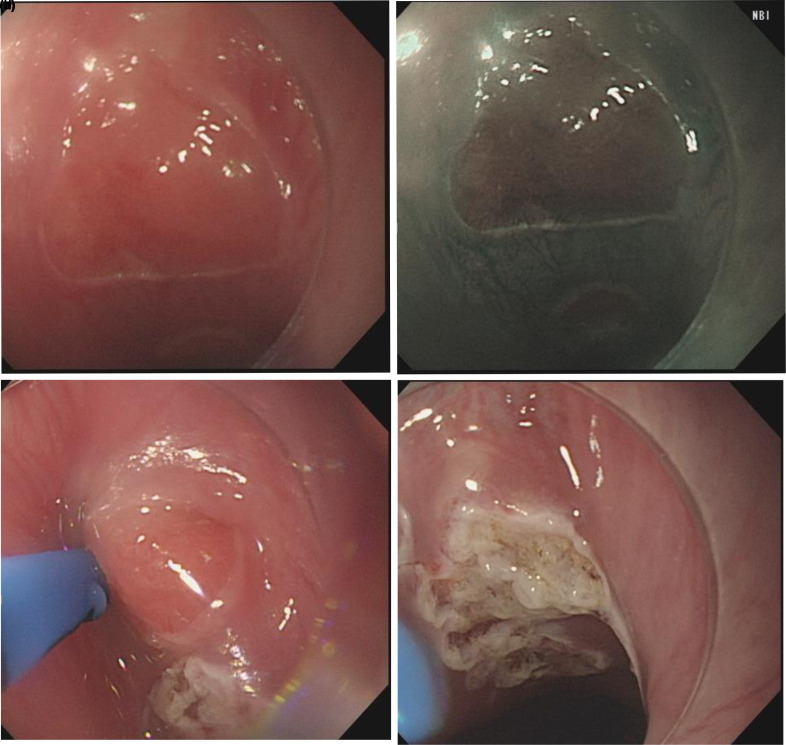
White-light endoscopy (A) and narrow-band imaging (B) demonstrating two salmon-colored patches measuring 0.8 cm and 0.4 cm at 18 cm and 20 cm from the incisors, respectively. Submucosal lifting with normal saline injection using the hybrid APC probe (C) was performed, followed by argon plasma coagulation ablation to the 0.8 cm patch (D).

## Data Availability

The data that support the findings of this study are available on request from the corresponding author.
