# Supplementary material for: The First Reported Case of Hybrid Argon Plasma Coagulation Ablation for Symptomatic Cervical Inlet Patches Refractory to Proton Pump Inhibitor Therapy
Source: Turk J Gastroenterol. 2026 Mar 8;37(6):732–4. doi: 10.5152/tjg.2026.25581 (PMC13247863; doi:10.5152/tjg.2026.25581)
Supplement: Supplementary Material [file supplementary_material.pdf]

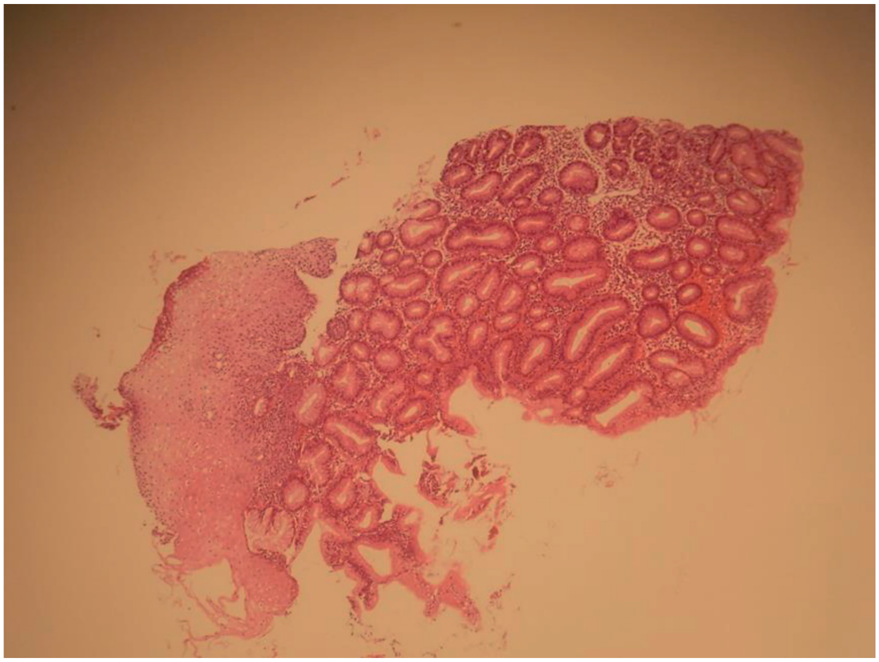

Supplementary Figure 1. Histopathology showing gastric-type mucosa adjacent to esophageal squamous epithelium with foveolar hyperplasia, consistent with cervical inlet patch (H&E, ×40).

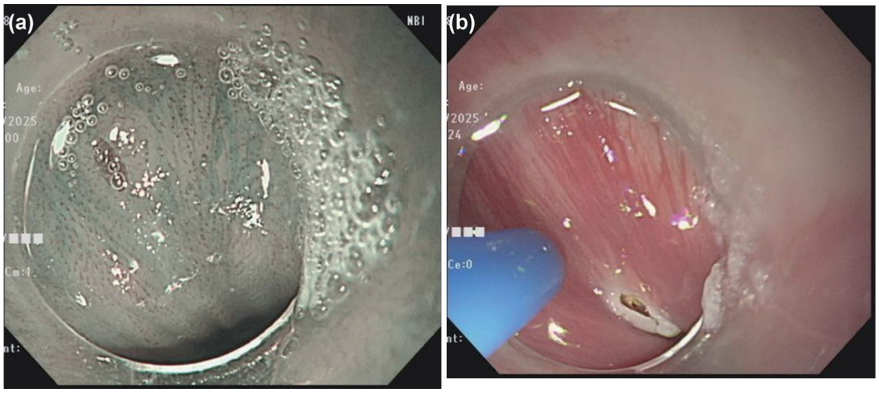

Supplementary Figure 2. Six-month follow-up endoscopy with narrow-band imaging demonstrating a small residual cervical inlet patch (~0.2 cm) at 18 cm from the incisors, treated with conventional argon plasma coagulation.
